# A comparison of conventional and retrospective measures of change in symptoms after elective surgery

**DOI:** 10.1186/1477-7525-9-23

**Published:** 2011-04-11

**Authors:** Eva M Bitzer, Marco Petrucci, Christoph Lorenz, Rugzan Hussein, Hans Dörning, Alf Trojan, Stefan Nickel

**Affiliations:** 1 ISEG Institute for Social medicine, Epidemiology, and Research in Health System, Lavesstr. 80, D-30159 Hannover, Germany; 2 University of Education, Dept. of Public Health and Health Education, Kunzenweg 21, D-79117 Freiburg, Germany; 3 Clinic of the Hamburg-Eppendorf University, Centre for Psychosocial Medicine, Institute for Social Medicine, Martinistraße 52, D-20246, Hamburg, Germany

## Abstract

**Background:**

Measuring change is fundamental to evaluations, health services research and quality management. To date, the Gold-Standard is the prospective assessment of pre- to postoperative change. However, this is not always possible (e.g. in emergencies). Instead a retrospective approach to the measurement of change is one alternative of potential validity. In this study, the Gold-Standard 'conventional' method was compared with two variations of the retrospective approach: a perceived-change design (model A) and a design that featured observed follow-up minus baseline recall (model B).

**Methods:**

In a prospective longitudinal observational study of 185 hernia patients and 130 laparoscopic cholecystectomy patients (T0: 7-8 days pre-operative; T1: 14 days post-operative and T2: 6 months post-operative) changes in symptoms (Hernia: 9 Items, Cholecystectomy: 8 Items) were assessed at the three time points by patients and the conventional method was compared to the two alternatives. Comparisons were made regarding the percentage of missing values per questionnaire item, correlation between conventional and retrospective measurements, and the degree to which retrospective measures either over- or underestimated changes and time-dependent effects.

**Results:**

Single item missing values in model A were more frequent than in model B (e.g. Hernia repair at T1: model A: 23.5%, model B: 7.9%. In all items and at both postoperative points of measurement, correlation of change between the conventional method and model B was higher than between the conventional method and model A. For both models A and B, correlation with the change calculated with the conventional method was higher at T1 than at T2. Compared to the conventional model both models A and B also overestimated symptom-change (i.e. improvement) with similar frequency, but the overestimation was higher in model A than in model B. In both models, overestimation was lower at T1 than at T2 and lower after hernia repair than after cholecystectomy.

**Conclusions:**

The retrospective method of measuring change was associated with a larger improvement in symptoms than was the conventional method. Retrospective assessment of change results in a more optimistic evaluation of improvement by patients than does the conventional method (at least for hernia repair and laparoscopic cholecystectomy).

## Background

Assessing quality of life is essential for evaluating health care services, quality management and policy making. Hence, it is important to accurately detect differences between patient groups and changes regarding different symptoms over time. Such differences and changes concern measuring change in pain, impairment and other symptoms associated with a specific condition. In this context, various approaches of measuring change have been presented. For example, the 'conventional' method and a 'retrospective' method. The conventional method consists of (at least) two points of assessment: preinterventional (pretest) and postinterventional. It is considered as the "Gold Standard" because the pretest enables the researchers to use a large number of statistical tests, which in turn facilitates measuring changes throughout the whole observation period. The conventional method is widely used in clinical studies [1]. However, there are situations where the application of this method is not possible, for example in unforeseen cases and emergencies, where collecting preoperative data is unfeasible. Moreover, the conventional method requires more efforts regarding organisation, logistics and costs compared to a retrospective alternative. In such cases, the retrospective approach, which assesses the patient's status only after intervention, can be more appropriate [1]. Two different models of retrospective measurement of change are applied in this study: the perceived change design (model A) and a design that featured observed follow-up minus baseline recall (model B). In model A, patients are required to report their status after intervention and to estimate the amount and/or direction of change, i.e. whether their condition has improved or worsened [2]. To date, only a few studies and even fewer German-language publications have considered the retrospective approach [3-6].

**Figure 1:**
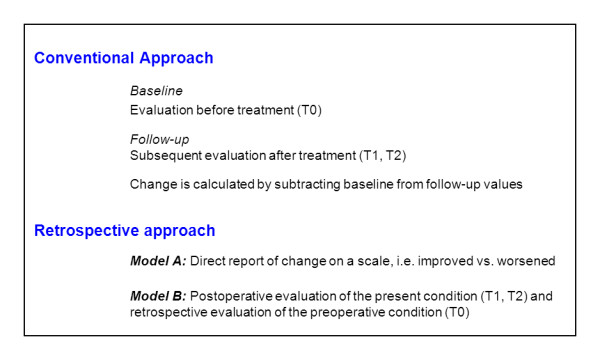
**Illustration of the models referred to in this article**.

Compared to model A, in model B, patients are asked about their present postoperative status and, retrospectively, about their preoperative condition. This retrospective re-evaluation is based on the assumption that patients will apply the same assessment criteria to the present follow-up as to the recalled baseline. This permits comparison between the two points of evaluation [7]. Figure 1 illustrates the models referred to in this article.

In spite of the above-mentioned advantages of the retrospective approach instead of the gold standard conventional method, there is a particular risk of recall bias. When interpreting findings of studies using this alternative method, recall bias must be taken into consideration and this may lead to over- or underestimation of the effectiveness of a treatment [8,9]. For example, researchers reported on retrospective overestimation of the effectiveness of low back pain surgery [10] and in lower urinary tract symptoms in patients with advanced prostate cancer [7]. Extent of recall bias can depend on the amount of time elapsed between intervention and data collection, but findings are equivocal. Marsh et al. found that older patients were able to accurately recall their preoperative health status at six weeks postoperatively [11]. Also, Bryant et al. found that patients undergoing knee surgery had no difficulty in recalling their preoperative quality of life, function, and general health at 2 weeks postoperative [12]. In contrast to these findings, Broderick et al. observed that rheuma patients had increasing difficulty remembering pain and fatigue symptom levels after as short as seven days [13]. Some researchers report that after a mean period of 2.5 years, patients had poor memory concerning their pain and function, and moderate recall of their walking ability [14]. In contrast, in a study conducted in Spain, recall time ranged between 2 and 58 months. This, however, did not affect the absolute agreement and consistency of the test used [10].

Additionally, Lam et al. found that model A is more susceptible to contamination by social desirability response bias than model B. However, Howard et al. found no differences in this regard between the two models [2,15].

Previous studies applied Model A to measuring change in areas of social functions [3], problems in psychosomatic rehabilitation [16] and instructional practice [2].

In this study, we measured patient-reported change in specific symptoms including pain and limitation of physical activity related to hernia repair and laparoscopic cholecystectomy before and after surgery. The aim was to compare the conventional method with two alternatives of the retrospective approach, i.e. the perceived change design (model A), and a design that featured observed follow-up minus baseline recall (model B). Our goal was to investigate the validity and acceptability of the two alternatives of the retrospective approach in comparison with the conventional procedure.

## Methods

### Study Design

We conducted a longitudinal study in two short-stay surgical units between August 1999 and January 2002. Data from patients with either hernia repair or laparoscopic cholecystectomy were collected using questionnaires at three points of measurement: 7-8 days preoperatively (T0), 14 days postoperatively (T1) and six months postoperatively (T2). Questionnaires used at T0 and T1 were handed out during the routine preoperative and postoperative visits by the treating surgeon. Questionnaires used at T2 were sent to the participants by mail by the surgical unit. Informed consent was obtained at T0.

For hernia, the realized three time points of survey were as follows: Eight days before surgery (T0), 13 days (T1), and six months after surgery (T2). The time points for gall bladder patients were seven days before surgery (T0), 11 days (T1) and six months after surgery (T2).

### Study Sample

Our study sample consisted of patients either with hernia repair (n = 185), or with laparoscopic cholecystectomy (n = 130). All patients filled out the standard questionnaire at baseline and follow-up (conventional approach). In addition, two thirds of our participants filled out the Model B questionnaires and one third filled out the Model A questionnaires at follow-up, respectively. 33.5% of patients with hernia and 20.8% of patients with gall bladder filled out the Model A questionnaires. Patients with hernia operation were mainly men (92.4%), mean age 58.6 years. About two thirds of the patients with gall bladder operation were women, mean age 53.6 years.

### Instruments

Indication-specific symptom checklists were used to assess symptoms preoperatively and postoperatively: The Hernia Symptoms Checklist (HSCL; [17]) consisting of nine items including difficulties bending forward, impairment in physical activities, groin pain, and numbness and the Gall Symptoms Checklist (GSCL; [18]; based on the gastrointestinal quality-of-life-index; [19]) with eight items including upper gastric pain, bloating, nausea and vomiting, loss of appetite and impairment in physical activity. The symptoms are rated on a four point scale (0 = no symptoms, 1 = little, 2 = moderate, 3 = strong). A total score is computed by summing up the single items. Scores range between 0 and 27 for HSCL and between 0 and 24 for GSCL, with a high score corresponding to high intensity of symptoms/impairment.

At T0, the preoperative status of all patients was assessed. They filled out a questionnaire containing questions regarding their current symptoms and a global rating of their symptoms, e.g. how strong their symptoms were before the surgery. The data thus collected were used as baseline values for the conventional measurement approach.

At T1 and T2, patients were asked about their current symptoms postoperatively. These data were used as follow-up values for the conventional measurement. In addition, the postoperative health status was also assessed with one of the alternatives of the retrospective measurement approach. The postoperative survey also included three questions regarding a global assessment of symptoms: "How strong are your symptoms?", "How strong were your symptoms before surgery?" and, "Has the severity of your symptoms changed compared to the time before surgery?". Approximately two thirds of the patients in our study (group 1) received the model B questionnaire for the two postoperative assessments, while the other third (group 2) received the model A questionnaire.

### Measuring Change

The conventional measurement of change in symptoms was implemented by subtracting the observed baseline values from the observed follow-up values. In model B, a measure of change was computed by subtracting the *recalled *baseline values from the observed follow-up values. In model A, we asked directly for the perceived amount of change. The interpretation of change in item values is illustrated in Table 1.

**Table 1:** Measuring change using the single items of the symptoms checklist

Method	Measuring change	Assessment points		Values*	Interpretation**
		**Baseline**	**Follow-up**		

Conventional	Δ follow-up - baseline	"How much pain do you have?"	"How much pain do you have?"	-2 to +2	< 0 = Decrease
	
Retrospective A	Perceived change***		"How much pain do you have compared to the time before the intervention?"	-2 to +2	0 = No change
	
Retrospective B	Δ follow-up - recalled baseline		"How much pain do you have?" "How much pain did you have before the intervention?"	-2 to +2	> 0 = Increase

Notes:

* The values '-3, -2, +2, +3' were summarised to '-2' or '+2', in order to have a direct comparison between the methods

** For all types of measurements of change

*** -2 = strong worsening, -1 = mild worsening, 0 = no change, +1 = mild improvement, +2 = strong improvement

### Clarification of the research aim

We were interested in examining the percentage of missing values and the strength of association between the methods. In addition, we wanted to know, whether the differences, i.e. overestimation and underestimation in both models of the retrospective approach compared to the conventional method are systematic.

Further questions concerning model B included:

• Is the recalled preoperative status (total score on symptoms list) systematically over- or underestimated?

• Does amount and direction of divergence (caused by over- or underestimation) depend from the severity of symptoms observed at baseline and follow-up?

• Do observed and recalled values differ systematically between the two diagnosis groups?

An analysis of validity was performed for both symptoms lists (hernia repair and laparoscopic cholecystectomy) and for the global assessment items.

### Statistical Analysis

Magnitude and direction of change were calculated for each item of the checklist for both indications (total scores for HSCL and GSCL) and for the global assessment of symptoms. Additionally, we examined the percentage of missing values for single items and the strength of association between the methods. Spearman's rank correlation coefficient (ρ), Kendall's tau b and Kappa statistics were used to examine the associations between conventional and retrospective values. Spearman's rank correlation and Kendall's tau b are non-parametric measures of association for ordinal scales. Their directionality indicates a positive or negative association, while their absolute values indicates the strength of the association. However, since our single items had a limited range of values, we also computed Kendall's tau- b because it uses a correction for ties [20]. The last measure of association we used was the unweighted Kappa. A Kappa > 0.4 indicates a moderate agreement, whereas a Kappa > 0.6 can be interpreted as good agreement [21].

## Results

### Missing Values

Missing values indicate the patient-acceptance of the different assessment methods. Missing values in model A were compared with those in model B at T1 and T2. Results showed that the amount of missing values in the former was higher in model A (Table 2).

**Table 2:** Single-Items Missing Values by Mode of Measurement Model and Time

Model	Point of measurement	Description	Average missing values	
			Hernia	Gall
Conventional, Subgroup A	T0	Measured directly	23,8%	20,8%
Conventional, Subgroup B	T0	Measured directly	24,2%	40,7%
A	T0	Perceived at T1	23,5%	33,3%
B	T0	Recalled at T1	7,9%	8,4%
A	T0	Perceived at T2	26,9%	33,3%
B	T0	Recalled at T2	8,9%	10,7%
A	T1	Measured directly	6,8%	10,7%
B	T1	Measured directly	11,1%	29,1%
A	T2	Measured directly	7,1%	9,3%
B	T2	Measured directly	14,9%	11,6%

### Correlation between conventional and retrospective data

As mentioned in the methods section, Spearman's ρ, Kendall's tau b and the unweighted Kappa statistic were all used to investigate the associations between conventional and retrospective data. Table 3 shows the degree of association between the amount of change resulting from the different models of measurement.

**Table 3:** Correlation between the Indirect and Direct Methods for Both Indications

	Spearman (ρ)				Kendell's tau b				(unweighted) Kappa coefficient*			
**Item**	**T1**		**T2**		**T1**		**T2**		**T1**		**T2**	

**Hernia**	**A**	**B**	**A**	**B**	**A**	**B**	**A**	**B**	**A**	**B**	**A**	**B**

b1	0.59	0.77	0.29	0.46	0.51	0.68	0.26	0.41	0.3	0.45	0,21	0,32
b2	0.47	0.73	0.1	0.57	0.4	0.65	0.09	0.53	0.15	0.5	0,13	0,42
b3	0.48	0.77	0.38	0.58	0.51	0.67	0.34	0.53	0.24	0.45	0,11	0,4
b4	0.45	0.76	0.47	0.41	0.39	0.67	0.43	0.37	0.18	0.41	0,26	0,22
b5	0.2	0.69	0.38	0.43	0.17	0.61	0.34	0.38	0.12	0.46	0,19	0,22
b6	0.39	0.63	0.29	0.47	0.33	0.56	0.25	0.41	0.2	0.43	0,21	0,25
b7	0.48	0.78	0.3	0.47	0.41	0.71	0.27	0.43	0.07	0.51	0,11	0,29
b8	0.49	0.64	0.21	0.46	0.42	0.56	0.17	0.4	0.16	0.36	0,08	0,24
b9	-0.01	0.37	0.08	0.22	-0.01	0.35	0.07	0.21	-0.003	0.34	-0,001	0,21
MW**	0.39	0.68	0.28	0.45	0.35	0.61	0.25	0.41	0.16	0.43	0,14	0,29
**GA°**	0.62	0.54	0.36	0.54	0.54	0.46	0.33	0.48	0.12	0.15	0,16	0,002

**Gall bladder**												

b1	-0.04	0.55	0.11	0.37	-0.02	0.49	0.11	0.34	-0.01	0.34	0,19	0,24
b2	-0.18	0.84	-0.04	0.51	-0.14	0.78	-0.03	0.44	0.05	0.59	0,16	0,21
b3	0.2	0.61	0.16	0.54	0.17	0.56	0.15	0.5	0.4	0.45	0,26	0,38
b4	0.27	0.68	0.52	0.35	0.22	0.64	0.51	0.32	0.3	0.53	0,28	0,19
b5	0.13	0.7	-0.11	0.43	0.1	0.64	-0.1	0.39	0	0.36	-0,02	0,16
b6	0.09	0.61	-0.04	0.34	0.08	0.53	-0.03	0.3	-0.02	0.33	0,02	0,08
b7	0.4	0.65	0.36	0.58	0.33	0.59	0.29	0.5	0.13	0.5	0,26	0,32
b8	0.05	0.66	0.14	0.47	0.04	0.58	0.13	0.42	0.01	0.26	0,08	0,21
MW**	0.12	0.66	0.14	0.45	0.1	0.6	0.13	0.4	0.11	0.42	0,15	0,22
**GA°**	0.31	0.44	0.36	0.4	0.27	0.38	0.33	0.35	0.06	0.04	0,12	-0,03

Notes:

A = Perceived change, B = Δ post - recalled T0

* Simple Kappa value

**MW: Mean correlation/ mean Kappa

° GB: Global assessment

# dichotomized differences in symptom values

Spearman's rank correlation coefficient showed that model B had a stronger association with the conventional assessment than did model A. This was true for both points of assessment, for both, hernia and gall bladder and for each single item. For example, the mean correlation at T1 of model A with the conventional method was 0.39 for hernia, while model B was correlated 0.68. Furthermore, correlation between conventional and both the retrospective alternatives was stronger at T1 than at T2. For example, for hernia patients, the mean correlation between model B and conventional measurement was 0.68 at T1 and 0.45 at T2. Compared to the global assessment items, correlation between the two alternative methods was less strong for each single item. With only one exception, model B showed a stronger relation to conventional assessment than did model A. With increasing time, the correlation between the global items decreased less than did the correlation between the respective single items.

Furthermore, we found indication-specific differences, i.e. the correlation of both retrospective models with the conventional method was stronger for gall bladder data than for hernia data, especially in model A.

As expected, Kendall's tau b also showed, the association between model B and conventional data to be positive. For both indications, this association was stronger on the level of single items than on the level of global assessment at T1. As a trend, with the elapse of time, the difference between global assessment and single items tended to decrease for both indications. A decrease in association between retrospective and conventional measurement from T1 to T2 was also observed.

Table 3 also shows that the degree of association between conventional assessment and model A was lower than between conventional assessment and model B (both in a negative direction).

The last measure of association used was the unweighted Kappa. The degree of agreement between model A and conventional assessment was lower than between model B and conventional assessment. For both models, the agreement was higher at T1 than at T2 and higher for the single items than for the global assessment items. For model A, the K-coefficient values did not exceed 0.3, which can be considered as low agreement [21].

### Overestimation and Underestimation of the T0 Measurement in Model B

This analysis was conducted with data from the conventional approach and from model B. It was not performed for model A because this analysis compares total scores that are not present in model A.

#### Changes in the symptoms sum score

The analysis was based on observed postoperative and recalled preoperative assessments. As shown in Table 4, the recalled values for both indications at T1 and T2 were higher than the observed values at T0. The increase in the symptoms sum score at T1 amounted to 6.1 points for hernia and 10.6 points for gall bladder. This could be seen as an overestimation of the severity of preoperative symptoms.

**Table 4:** Preoperative Total Scores Model A and Model B and Their Correlation

	Hernia (n = 120)			Gall Bladder (n = 95)		
**Preoperative checklist**	**Observed**	**Recalled**	**Recalled**	**Observed**	**Recalled**	**Recalled**
**(Total scores)**	**at T0**	**at T1**	**at T2**	**at T0**	**at T1**	**at T2**

Preoperative checklist	30,7	36,8	41,2	30,7	41,3	48
Δ T0 recalled - T0 observed		6,1	10,5		10,6	17,3
Correlation with T0 observed						
Spearman **(ρ)**		0,73	0,61		0,65	0,53
Kendel'sτb		0,59	0,46		0,51	0,4
Correlation with Post						
Spearman		0,09	0,13		0,43	0,29
Kendel'sτb		0,06	0,1		0,31	0,22

#### Correlations between observed and recalled symptoms scores

The recalled values of the preoperative symptoms had a higher correlation with the observed T0-values than with the current postoperative total scores of the checklist. For hernia, the former was 0.73 and the latter was 0.09. In general, the recalled preoperative symptom values had stronger associations with the observed value at T0 than with the respective postoperative value.

The overestimation in the recalled values of the preoperative symptoms was higher for gall bladder than for hernia patients, while the degree of association with the observed T0-values of the symptoms list was higher for hernia than for gall bladder. Yet, the association with the postoperative value of the symptoms list was higher for gall bladder (0.43) than for hernia (0.09). There was an increase of 11.2 points in the observed-symptoms score for hernia patients at T1. The increase in the recalled- symptoms score was 5.1 points, which means that the postoperative worsening of symptoms was underestimated by about 6.1 points. This did not apply to the T2 data. The improvement of symptoms (mean = 20.6 points) was overestimated by an average of 10.5 points compared to the values observed postoperatively.

In gall bladder patients, there was an even higher overestimation of improvement at both, T1 and T2 (Table 5).

**Table 5:** Difference of Total Scores of the Checklists for Conventional and Retrospective Measurement (Model B)

	Hernia (n = 120)				Gall Bladder (n = 95)			
	**Δ T1°**		**Δ T2°**		**Δ T1°**		**Δ T2°**	

	**Observed**	**Recalled**	**Observed**	**Recalled**	**Observed**	**Recalled**	**Observed**	**Recalled**
Difference	+11.2	+5.1	-20.6	-31.1	-2.2	-12.7	-15.5	-32.8

#### The effect of the observed preoperative or postoperative value on the overestimation of the recalled preoperative values

This examination was carried out by stratifying the observed postoperative and recalled preoperative data according to the level of the observed preoperative values into high or low level of symptoms. As can be seen in Table 6, patients who had a low observed preoperative value at T0 overestimated the severity of their postoperative symptoms, compared to patients with high observed preoperative value at T0. For example, patients with hernia operation who had less symptoms preoperatively compared to the other subgroups overestimated their symptoms by an average of 9.0 points, whereas those with high preoperative values overestimated their symptoms only by an average of 2.4 points. In contrast, we observed, that, in hernia patients with low observed symptoms at T2, there was a similar overestimation in symptoms compared to the subgroup with high observed symptom scores at T2 (6.7 vs. 5.6).

**Table 6:** Level of Recalled Preoperative Complaints Depending on the Observed Level of Complaints at Different Time Points

Preoperative checklist total scores	Observed value at T0		Observed value at T1		Observed value at T2	
	**Low**	**High**	**Low**	**High**	**Low**	**High**
**Hernia (n = 120)**	**≤ 30**	**> 30**	**≤ 30**	**> 30**	**≤ 4**	**> 4**

Observed	16.0	49.3	29.1	32.0	27.8	33.2
Recalled T1	25.0	51.7	35.4	37.9	34.6	38.8
Recalled T2	32.0	52.9	38.7	43.1	38.0	44.0

Δ recalled T1 - observed T0	+9.0	+2.4	+6.3	+5.9	+6.7	+5.6
Δ recalled T2 - observed T0	+16.0	+3.6	+9.7	+11.2	+10.2	+10.8

	**Low**	**High**	**Low**	**High**	**Low**	**High**
**Gall bladder (n = 95)**	**≤ 28**	**> 28**	**≤ 28**	**> 28**	**≤ 10**	**> 10**

Observed	14.8	45.1	26.2	35.2	26.1	35.0
Recalled T1	29.7	51.7	33.8	48.6	36.6	45.7
Recalled T2	39.2	56.0	43.5	52.4	42.7	53.1

Δ recalled T1 - observed T0	+14.9	+6.6	+7.6	+13.4	+10.4	+10.6
Δ recalled T2 - observed T0	+24.4	+10.9	+17.4	+17.2	+16.5	+16.7

At T1/T2, there was also an overestimation of symptom severity for both indications though it did not depend on the level of postoperative symptoms (high vs. low). We observed that the difference between values at T0 and T1/T2 that depended on the level of postoperative symptoms was constant over time. The only exception was in gall bladder patients with low observed postoperative symptoms at T1, who had less overestimation of the recalled preoperative values compared to those with high observed postoperative values (7.6 vs. 13.4 points) at T1. In summary, we conclude that the recalled preoperative values were overestimated more often if the observed preoperative values were low.

## Discussion

The "gold-standard', conventional method of prospective measuring change was associated with a large improvement of symptoms after elective surgery. However, for both hernia and cholecystectomy both retrospective approaches revealed even larger improvements. The two alternatives of the retrospective method overestimated the success of the surgical intervention compared to the conventional method. This overestimation of effectiveness increased with increasing time elapsed after the operation, i.e., overestimation was lower shortly after operation compared to six months afterwards. Our data confirm that the retrospective measurement of change that was a feature of model B, where pre-operative symptoms are collected retrospectively, is closer to the conventional baseline-follow-up measurement.

Memory represents a major concern in approaches depending on recalled data. The recall period may affect the agreement between prospective and recalled data. High association between retrospectively and prospectively collected data was observed by Singer et al. for an interval of 1 to 7 days between initial episode and assessment [22].

Recall may be better for some factors than for others. Better recall might be expected for physical function than for pain status because specific questions are answered more reliably [23]. Dawson et al. reported that radicular symptoms, frequency and location of pain and the way activities affect pain were recalled with greater accuracy than were the qualities of pain, e.g. severity [24]. Recall might be also influenced by patient characteristics including age, gender, surgery-expectations and the current status of pain and physical functioning [24]. Poorer recollection of physical function was reported in patients whose function scores had worsened three months after knee surgery [9]. Furthermore, patients with good mental health had similar pain memory compared to patients with poor mental health but the latter had significantly worse function recall [9]. Yet, another study in which poor agreements between retrospective and prospective data were found for both, pain and function scales, neither age nor gender nor current medical status modified the absolute agreement and consistency of the test being used [10].

Some researchers interpret differences between actual and recalled preoperative values as a change in the internal standards of a patient (response shift, [25,26]). A recent study [27] found that patients who underwent laparoscopic cholecystectomy reported a significantly higher 'Quality of Life' when asked directly before the operation, compared to the retrospective rating of their preoperative 'Quality of Life', which is interpreted as positive response shift. These results are in line with our findings concerning Model B.

Model A is also known as an anchorbased method frequently applied in research on determining the smallest patient reported outcome score difference that can be judged as meaningful [26] In our study, patients judged their situation as "improved" even when the conventional method showed modest worsening of symptoms (cholecystectomy T1 assessment). We think this finding is partly due to the intervention "elective surgical procedure": In the light of having "survived surgery" patient reported improvement might be reflective of an overall feeling of relief. Given this, minimal important changes after elective surgery assessed with anchorbased methods might be treated with caution.

In our study, our expected associations were found for both indications. Yet, these associations were sometimes less apparent in laparoscopic cholecystectomy patients. This may be due to indication-specific reasons, the very small sample size for Model A in cholecystectomy patients, or to the uneven distribution of men and women in the two samples (i.e. hernia patients were mainly male while gall patients were mainly female). This mismatch in distribution regarding gender made it difficult to check causes for the observed results unambiguously.

Model B represents a mixture of both the conventional and the retrospective perceived change approaches to measuring change in symptoms. In this study, we also observed that the values gained through model B were more similar to those gained through conventional measurement regarding the overestimation of symptoms than were the values gained through model A. This dual role of pro- and retrospective measurement is consistent with comments from other researchers that have warned of only depending on retrospectively collected data to determine preoperative status. It must be clear that such data is not a direct substitute for prospectively collected data. Because of the variable reliability in recalled data, there is the possibility that the effectiveness of interventions may be over- or underestimated [9]. However, in our study, we found an overestimation effect for both surgical interventions. Hence, retrospective measurement of change yielded more optimistic results than conventional assessment.

Our study has some limitations. First, our sample size was relatively small. This made it impossible to control for gender as a possible confounder (hernia repair affecting mainly men and laparoscopic cholecystectomy mainly women). Second, due to organisational constraints (i.e. difficulties in distributing the questionnaires in surgical units), more model B patients measured change through model B than model A (Model A was used by one third less patients). These two biases complicate the interpretation of our results. Therefore, it would be useful to undertake further research with larger numbers of cases and other indications. Nevertheless, we find it encouraging that data from such unequal samples led to consistent results.

## Conclusions

In both models relying on retrospective recall, the observed changes in the direction of improvement were larger than were the changes measured by the conventional method. As a conclusion, retrospective assessment of change results in a more optimistic evaluation of self-improvement than does the conventional method (at least for hernia repair and laparoscopic cholecystectomy).

## Competing interests

The authors declare that they have no competing interests.

## Authors' contributions

EMB was responsible for designing the study, analyzing the data, interpreting the findings, in addition to writing the paper and commenting on the drafts. CL was responsible for data analysis, interpretation of findings and commenting on the drafts of the paper. HD participated in study design and subsequent analysis and interpretation of data, in addition to drafting the manuscript. AT was involved in the design of the study, interpretation of findings, as well as commenting on the drafts of the paper. SN was responsible for designing the study, collecting the data, interpreting the findings, and commenting on drafts of the paper.

RJH participated in the interpretation of data, writing the paper and commenting on the drafts of the manuscript. MP participated in the interpretation of data, writing the paper and commenting on the drafts of the manuscript. All authors approved the final manuscript.
